# Terahertz Vibrational Dynamics and DFT Calculations for the Quantum Spin Chain Linarite, PbCuSO_4_(OH)_2_

**DOI:** 10.1021/acs.jpca.3c06926

**Published:** 2024-02-28

**Authors:** Andrew Squires, Evan Constable, Joseph Horvat, Dominique Appadoo, Ruth Plathe, R. A. Lewis, Kirrily C. Rule

**Affiliations:** † University of Wollongong, Wollongong, NSW 2522, Australia; ‡ Commonwealth Scientific and Industrial Research Organisation (CSIRO), Lindfield, NSW 2070, Australia; § Institute of Solid State Physics, TU Wien, 1040 Vienna, Austria; ∥ Australian Synchrotron, ANSTO, 800 Blackburn Rd Clayton, VIC 3168, Australia; ⊥ Australian Centre for Neutron Scattering, ANSTO, Lucas Heights, NSW 2234, Australia

## Abstract

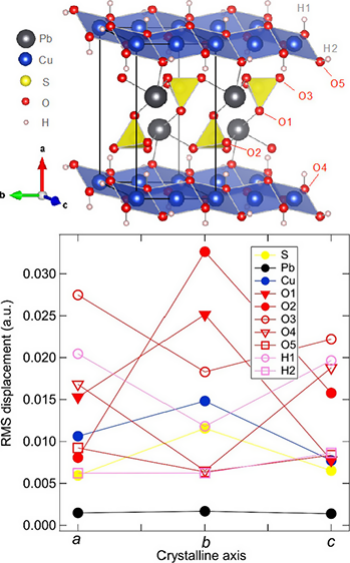

The low-dimensional quantum-magnet, linarite, PbCuS_4_(OH)_2_, has been investigated using terahertz (THz) spectroscopy coupled with detailed density functional theory (DFT) calculations in order to explore the effects of the temperature on its lattice vibrations. Linarite is characterized by largely isolated CuO chains propagating along the crystallographic *b*-axis, which at very low temperatures are responsible for exotic, quasi-1D magnetism in this material. To better understand the synergy between the structural bonds and lattice oscillations that contribute to these chains, polarized THz spectroscopic measurements were performed. Consolidating these results with detailed DFT calculations has revealed that the anisotropic vibrational motion for the THz modes is correlated with extreme motion associated with the crystallographic *b*-axis. An unexpected feature observed in the infrared spectrum is attributed to subtle lattice distortions which break the centro-symmetry in linarite at high temperatures. This phenomenon has not previously been observed in linarite and likely results from anharmonicity in lattice oscillations.

## Introduction

Linarite, PbCuS_4_(OH)_2_, is a naturally occurring copper-oxide mineral that forms needle-like single crystals through a process of weathering of lead and copper ore deposits.^1,2^ Natural minerals are interesting as they often form large, perfect single-crystal samples, which cannot be grown in a lab but can provide a suitable matrix for investigating a range of unusual properties.

**Figure 1:**
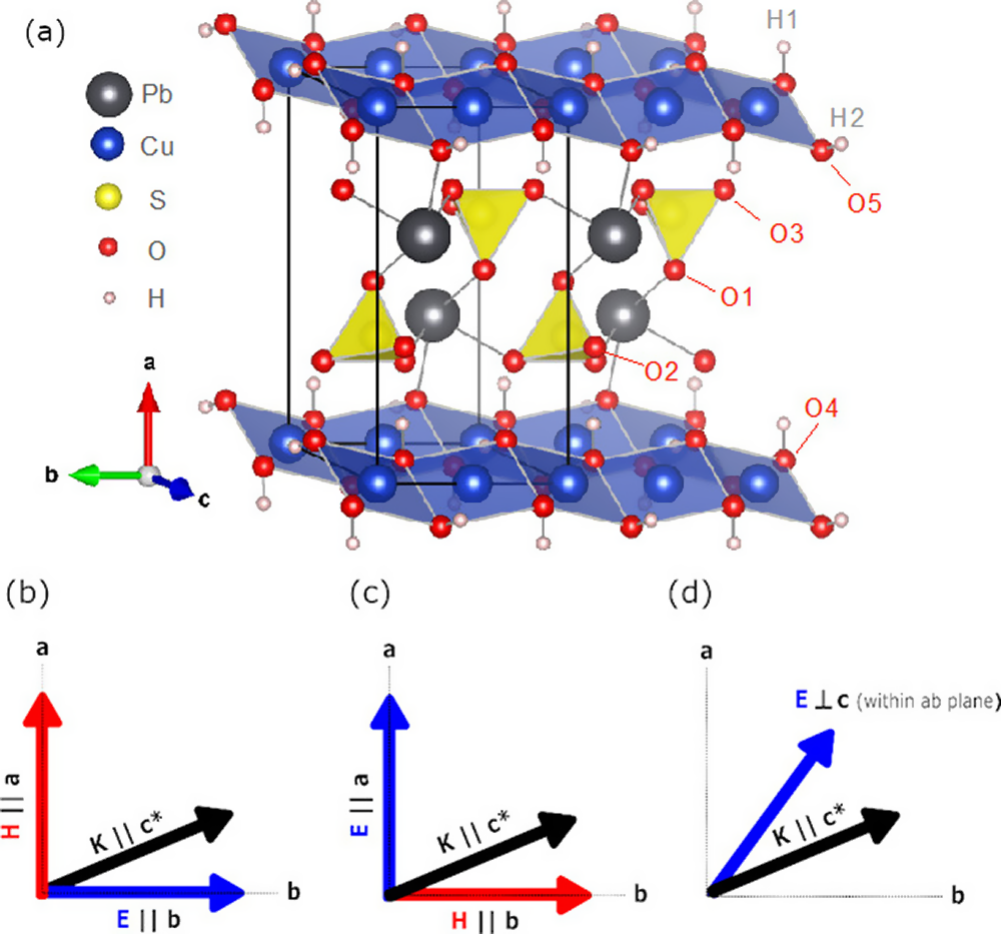
(a) Crystal structure of linarite where dark gray spheres are Pb, yellow are S, red are O, blue are Cu, and white are H. Experimental conditions for the THz measurements (b–d) with the directions of applied electric polarization, *E*, relative to the crystallographic axes. In all instances, propagation of the terahertz wave, *K*, was oriented along the crystallographic *c*-axis.

Linarite is one such material that hosts unusual properties including a frustrated, incommensurate magnetic ground-state and multiferroic behavior (Figure 1a).^3−9^ Such properties are important components of new models that describe high-temperature superconductivity and multiferroicity.^8,9^ In linarite, the Cu^2+^ ions form spin s = 1/2 chains along the *b* direction, which form a magnetically frustrated topology that enters a helical magnetically ordered state below *T*_N_ = 2.8 K.^3,4^

Linarite crystallizes in the monoclinic centrosymmetric space group *P2*_*1*_/*m* (International Table for Crystallography (ITC) space group #11, with *b* being the unique axis) and forms a needle-like growth morphology. The lattice parameters of linarite (at 1.8 K) are *a* = 9.682 Å, *b* = 5.646 Å, *c* = 4.683 Å, and β = 102.65°, with the longest direction of the needle-like crystal being along the crystallographic *b*-axis—the same axis as the strongest magnetic interactions in the spin chain.^3^ The recent interest in linarite can be attributed to the delicate balance among magnetic interactions, frustration, and quantum spin effects. This complex interplay has promoted linarite as a strong candidate to display spin nematic behavior at low temperatures in applied fields close to the saturation field.^4−7^

There has also been recent speculation as to how electronic effects might couple to the magnetism in linarite, revealing multiferroic-type behavior.^8,9^ In fact, the elliptical spin structure of the ground state, which is incommensurate with the crystal lattice, is ideal for supporting multiferroicity, with indications that a ferroelectric transition coincides with the onset of long-range magnetic order. In the work by Yasui et al.,^8^ an anomaly in the dielectric constant indicated a second-order, direct transition to a multiferroic phase when an electric field was applied parallel to the a*-axis of a single-crystal sample (corresponding to the [100] direction in reciprocal space). This was further supported by electric polarization measurements, which showed a bias-induced electric polarization in powder samples of linarite when the electric field was reversed during cooling.^8^

One theory of the origin of the improper ferroelectric behavior involved possible unnoticed structural variations, which may occur at higher temperatures. This structural variation suggests a potential loss of inversion symmetry, which may occur due to spin–phonon coupling. Weak structural changes have been reported to similarly affect the ground-state magnetic behavior in another material, NTENP.^10^ In that work, it was believed that a weak structural phase transition around 170 K could allow for additional anisotropies (such as Dzyaloshinskii Moriya (DM) or in-plane interactions) to manifest themselves in the low-temperature regime, evoking a staggered magnetic field, which could result in the observed finite energy gap. Structural changes were also reported to influence the ground-state magnetism in the frustrated kagome francisites, namely, Cu_3_Bi(SeO_3_)_2_O_2_*X*, where *X* = Cl, Br, or I.^11^ In this material, a spin gap in the ground state was also attributed to anisotropies which arise concurrently with a structural distortion at moderate temperatures. Both examples given here indicate a strong interplay between structure and magnetism in low-dimensional and frustrated magnets, which may also contribute to the multiferroic properties of these materials. While no spin gap has been observed in linarite,^5^ the relative contributions of the structure, magnetism, and ferroelectricity may be critical to the observed behavior.

It is this interplay that has motivated the current study: to look for a link between structural and magnetic excitations. Since the low-energy magnetic excitations across multiple Brillouin zones have been studied with inelastic neutron scattering techniques,^4−7^ this study focuses more on the Brillouin zone center (optical) excitations up to very high energies. One method for investigating the phonon excitations and lattice dynamics in linarite is far-infrared, or terahertz, spectroscopy. By monitoring the phonon frequency and absorption strength with changing temperature and radiation orientation, new magnetoelectric interactions may be revealed in the complex mineral system.^12^ These results may reveal the role of structural variations and loss of centro-symmetry on the behavior of linarite. Combining these results with ab initio density functional theory (DFT) calculations should provide an understanding of the eigenvectors for the vibrational modes and reveal the local dynamic distortions that characterize them. Moreover, the presence of new modes would indicate a global structural distortion. These results are important as the appearance/disappearance of phonons can reveal structural transitions which may be missed in X-ray or neutron diffraction experiments if the transition is between subgroups with identical extinction classes.

## Experimental Details

This work presents terahertz transmission spectroscopy on a single crystal of PbCuSO_4_(OH)_2_ (linarite) using synchrotron radiation. This is a continuation of the work published recently,^13^ in which data were collected over a limited temperature and polarization range. The current study covers the spectral region 150–400 cm^–1^ (∼18–50 meV, 4–12 THz) with electric field polarization parallel to either the *a* or *b* crystal directions. In this spectral range, the linarite samples, from the same source as those measured in refs (4 and 5), have a low optical transmission of ∼5–8%. Therefore, typical THz sources such as mercury lamps, globars, and photoconductive antennas are generally too weak to obtain meaningful transmission data, especially at higher frequencies above 150 cm^–1^. Moreover, as linarite is a naturally occurring crystal grown under extreme weathering processes,^1,14^ only small samples (mm scale) with rough surfaces can be obtained, limiting the possibility of performing reflectivity measurements. Therefore, we utilize the high incident flux of a synchrotron source in transmission to probe the phonon spectra of the small single-crystal linarite samples.

The terahertz measurements were performed using the ANSTO Terahertz-Far-Infrared beamline at the Australian Centre for Synchrotron Science.^15^ Spectra were acquired by using a Bruker IFS 125/HR Fourier transform infrared (FTIR) spectrometer. A 6 μm multilayer mylar beamsplitter was employed giving a spectral bandwidth of 30–630 cm^–1^ (∼4–80 meV, ∼1–20 THz). A helium-cooled Si bolometer was used for detection.

For spectral acquisition, 200 rapid scans were averaged at a 2 cm^–1^ resolution. To achieve temperature dependence, a closed-cycle pulse-tube cryostat with a base temperature of 6 K was coupled directly to the Bruker IFS 125/HR spectrometer. The impact from water absorption on the beam profile was minimized by using a high-vacuum sample chamber. Single-crystal linarite samples were chosen by visual inspection, where the most appropriate sample was ∼3 mm × 5 mm × 1 mm offering suitable transparency. From the same source as crystals in refs (4 and 5), this sample has been characterized using a variety of bulk probes including neutron diffraction and inelastic neutron scattering which confirm the high-quality single crystalline nature. Samples were mounted onto copper plates with a 5 mm diameter aperture that were attached directly to the coldfinger of the cryostat. This ensured good thermal contact between the sample and coldfinger of the cryostat. With this setup, temperatures down to 7.5 K were achieved with measurements taken up to 300 K. At each temperature, a minimum wait time of 10 min was allowed to ensure thermal equilibrium between the sample, temperature sensor, and coldfinger.

Polarization of the beam was controlled via a gold wire grid polarizer externally mounted to the cryostat window. Due to the highly elliptical polarization of the synchrotron beam, favored toward the horizontal axis, the polarizer was oriented to ensure the transmission of horizontally polarized light. The crystal orientation relative to the polarizer was manipulated by rotating the sample to guarantee that sufficient signal strength could be maintained. Rotating the polarizer resulted in a significant decrease in signal. In this configuration, electric field orientations of *E* || *a* (∼*H* || *b*) and ∼*E* || *b* (∼*H* || *a*) were achieved as outlined in Figure 1b–d. Due to the geometry of the crystal, we could not measure *E* || *c*.

Measurements were also performed on this material using a lab-based FTIR setup located at the University of Wollongong, Australia. These measurements were made at 1.6 K and in the presence of applied magnetic fields of up to 5 T using an integrated superconducting magnet. No significant changes to the absorption spectra were observed either within the magnetically ordered state or in the presence of an applied magnetic field, indicating that the observed features are most likely of phononic origin (the results of these measurements are provided in the Supporting Information).

DFT modeling was performed using the Quantum Espresso package.^16^ The PBESol DFT functional was used,^17^ with fully relativistic ultrasoft pseudopotentials.^18^ The kinetic energy and charge density cutoffs were 90 and 380 Ry, respectively. The Monkhorst–Pack integration grid was of 8 × 8 × 4 size. A nonmagnetic calculation with spin–orbit coupling was used with variable-cell geometry optimization. Preliminary measured spectra did not change when crossing the temperature of magnetic ordering, and the magnetic field up to 5 T did not affect the spectra below the ordering temperature (Supporting Information), which justifies the use of nonmagnetic calculation. As modeling was performed to obtain the infrared spectrum at low energies, very tight geometry optimization was needed to ensure that the modeled spectrum was similar to the experimental one. The optimization was considered satisfactory when the total residual force for the 22 atoms in the unit cell reached 10^–6^ Ry/bohr. Furthermore, the maximum Cartesian component of the residual force acting on an individual atom was 6.3 × 10^–7^ Ry/bohr. The phonon modes were calculated in the harmonic approximation without thermal effects. The Avogadro software package^19^ was used for visualization of the obtained normal modes of vibration.

## Results

**Figure 2:**
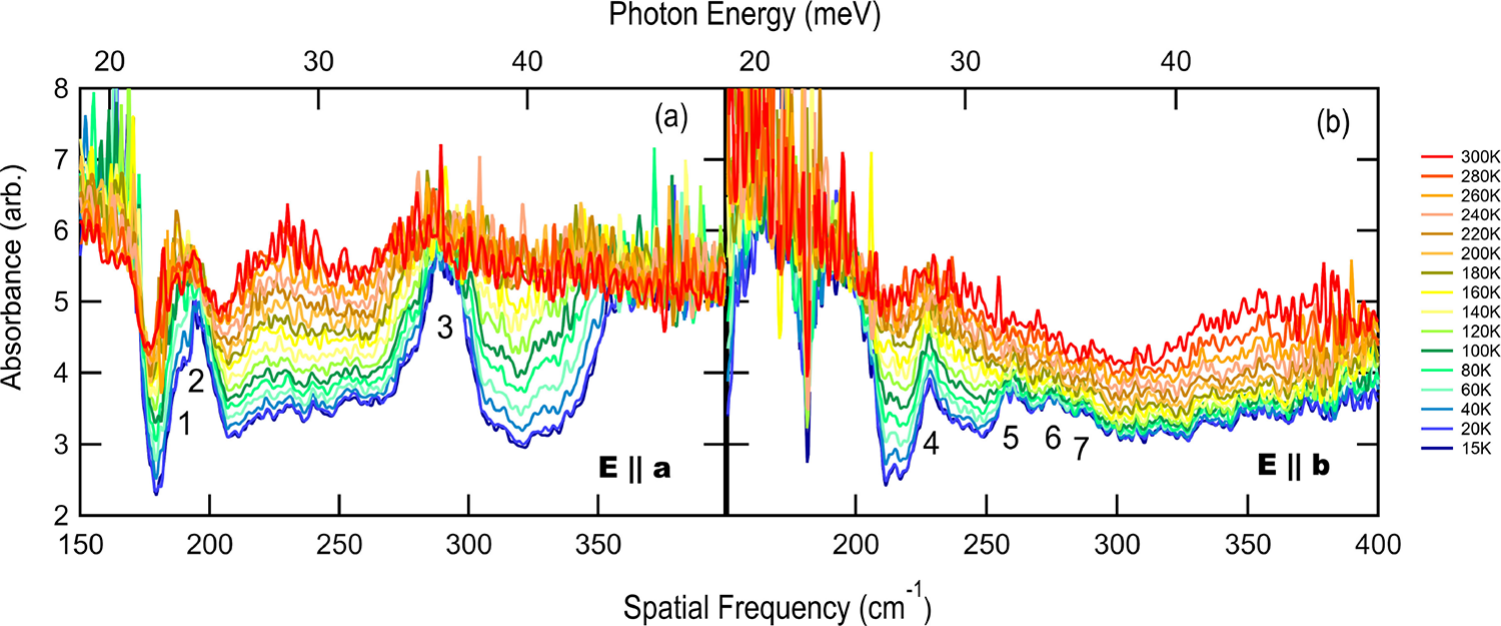
Optical absorbance (−ln(*T*), where *T* is transmission) in single crystalline linarite for electric polarization parallel to the *a*-axis (a) and the *b*-axis (b) as a function of temperature. The light propagates along the *c*-axis. Red indicates room temperature measurements, while blue data were collected at the lowest measured temperature of 15 K. The number labels embedded within the graph indicate the key absorbance features in each data set. We note that the axis labels in the previous conference publication^13^ were mislabeled, and this figure shows the correct electric field polarization axis.

Temperature-dependent measurements were taken for the electric polarization of the synchrotron beam applied parallel to either the crystallographic *a*-axis or *b*-axis—that is, either perpendicular or parallel to the Cu–O chain direction, respectively (these data can be seen in Figure 2a,b). From this figure, multiple, unique phonon absorption features were observed for each polarization direction. In general, the features do not shift significantly in energy with increasing temperature (see the Supporting Information), but they do appear to change intensity with temperature. The modes that disappear completely at higher temperatures can be attributed to thermal broadening, which leads to an overall smearing out of the signal at high temperatures when many of the accessible phonon modes are fully populated. This is typical behavior for these types of features.

The observed peaks in Figure 2b (*E* || *b*) between 220 and 300 cm^–1^ are highly anisotropic and do not appear in Figure 2a for *E* || *a*. For lower energies (<210 cm^–1^), the data also exhibit large isotropic absorption features for both polarization directions (*E* |*|b* and *E* || *a*) at around 175 and 194 cm^–1^. Two broad absorptions were observed for *E* || *a* at 290 cm^–1^ and above 348 cm^–1^, while from 220–260 cm^–1^, several possible (much weaker) features are observed including a feature centered at ∼230 cm^–1^ resembling a hot band, as will be discussed in detail below.

**Figure 3:**
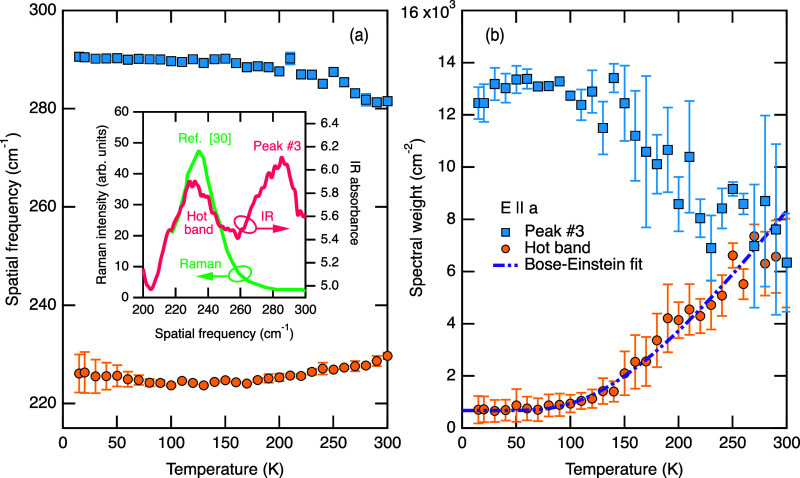
Temperature dependence of the peak position (a) and spectral weight (b) for the hot band and phonon mode #3 for the *E***||***a* spectra. Values are determined by a statistical average of several fits of the data in Figure 2a using Gaussian peak profiles and different background absorbance treatments. The spectral weight is approximated by a product of the peak position, amplitude, and full width at half-maximum. The error bars indicate standard deviations. The inset depicts a smoothed extract from the spectrum in Figure 2a at 300 K along with a Raman spectrum adapted from ref (23).

Due to their temperature-dependent behavior and presence above the magnetic ordering transition, which is at 2.8 K, the absorption bands observed above 15 K in Figure 2 can be attributed primarily to phonon modes: lattice vibrations and molecular rotations and torsions. Detailed fitting parameters of the associated absorption bands are included in the Supporting Information for reference. The intensity of each absorption band varies (sometimes quite significantly) with temperature. In general, their intensity decreases with increasing temperature. An exception to this trend is observed in Figure 2a, for *E* || *a*, where a broad absorption band between 210 and 250 cm^–1^ is weakest at 15 K but evolves into a strong absorption by 300 K. This behavior is depicted in Figure 3, where the fitted peak central frequency and spectral weight of this “hot band” are shown relative to the phonon designated #3 in Figure 2a. In contrast, the same spectral region for *E* || *b* shows several sharp, yet weak features, which become strongest below 100 K (labeled #4 in Figure 2b; see also the Supporting Information).

## Discussion

The appearance of the hot band may be attributed to several possible phenomena. Transitions between two excited crystal-electric-field levels separated by ∼230 cm^–1^ can produce such spectral features, as the lower of the two levels becomes populated at higher temperatures, allowing photons to drive transitions between the two states.^20^ In this case, fitting the temperature dependence of the spectral weight with a simple two-level Boltzmann model, as in ref (21), predicts the energy of the lower level to be ∼450 ± 50 cm^–1^.

Alternatively, the hot band may be the result of a mulitphonon difference band, in which a photon and a phonon of lower energy combine to excite a phonon of higher energy, resulting in an absorption band at the difference energy of the two phonons.^22^ Such excitations are driven by anharmonic processes and follow a characteristic temperature dependence as described in ref (22). Here, the temperature-dependent model of absorbed power fitted to the spectral weight suggests that the hot band could be due to a difference between a phonon band with an energy of 380 ± 60 cm^–1^ and one with an energy of 610 ± 60 cm^–1^. Such transitions typically occur between phonons at the zone boundary where the dispersion bands tend to be flat and the density of states is highest.

Perhaps a more intriguing possibility is that the hot band is a result of a normally infrared-silent mode becoming infrared-active at higher temperatures due to local symmetry breaking as a result of anharmonic lattice fluctuations. This possibility is supported by the presence of a Raman-active mode at ∼230 cm^–1^ as identified in ref (23) and depicted in the inset of Figure 3a. Moreover, there appears to be a clear transfer of spectral weight between phonon #3 in Figure 2a and the hot band above ∼100 K, accompanied by subtle shifts in the frequency position (Figure 3a), which is typically a sign of anharmonicity due to phonon–phonon scattering processes.^24^ The temperature dependence of the hot-band spectral weight is consistent with the Bose–Einstein statistics of a phonon population with an energy of 330 ± 60 cm^–1^, as obtained from the Bose–Einstein fit depicted in Figure 3b. Notably, this energy is higher than the central frequency of the hot band at 230 cm^–1^ but overlaps with the frequency of phonon #3. This suggests that it is the population of this phonon that drives the spectral intensity of the hot band. For these reasons, we propose that the most likely mechanism for the appearance of the hot band at 230 cm^–1^ is the infrared activation of a Raman-active mode involving an interaction with phonon #3 for the *E* || *a* orientation.

The implication of a Raman-active mode becoming infrared-active is a loss of centro-symmetry in the lattice, which causes the exclusivity of the Raman and infrared selection rules to be lifted.^25−28^ There are two possible noncentrosymmetric subgroups to which the *P2*_*1*_/*m* space group of linarite can directly transform. These are *P2*_*1*_ (ITC #4) and *Pm* (ITC #6), with *P2*_*1*_ being the more likely, as it involves a simple splitting of the 4*f* Wyckoff position occupied by the O3 ions that contribute to the Pb–O–S bonds along the *b* direction. Any distortion involving a net polar displacement along the *b* direction will break the *b*-axis mirror symmetry but maintain the 2-fold screw axis, while promoting a spontaneous *b*-axis polar moment.

Despite numerous diffraction studies of linarite single crystals,^1,2,29−31^ there have been no reports of a global symmetry descent from *P2*_*1*_/*m* to *P2*_*1*_ at close to room temperature. However, note that the reflection conditions of these two space groups are equivalent, with the only way to distinguish them being the refined number of Wyckoff positions. This means a very subtle *b*-axis distortion lowering the symmetry from *P2*_*1*_/*m* to *P2*_*1*_, perhaps involving localized domains or a long-range incommensurate periodicity, would likely give almost identical refinements for the crystal structure. This could easily be overlooked in the diffraction analysis. The onset of such distortions at high temperatures could be a result of anharmonic lattice dynamics or pinned charge carriers in the CuO_4_ chains forming localized polarons that create a short-ranged symmetry descent in the lattice. Similar mechanisms have been proposed to explain the dynamic symmetry breaking that allows for enhanced charge transport in photovoltaic lead-halide perovskites.^32,33^

To better understand the relationship between the molecular vibrations in the linarite lattice, computational phonon analysis is required. Thus, ab initio calculations were performed to provide eigenvectors for the vibrational modes to determine which parts of the lattice are moving for each normal mode excitation. To interpret spectral features from both the *a* and *b* crystallographic axes, data were also collected at an angle of 45° to the *a*-axis to compare with the DFT calculations, which are polarization-independent.

**Figure 4:**
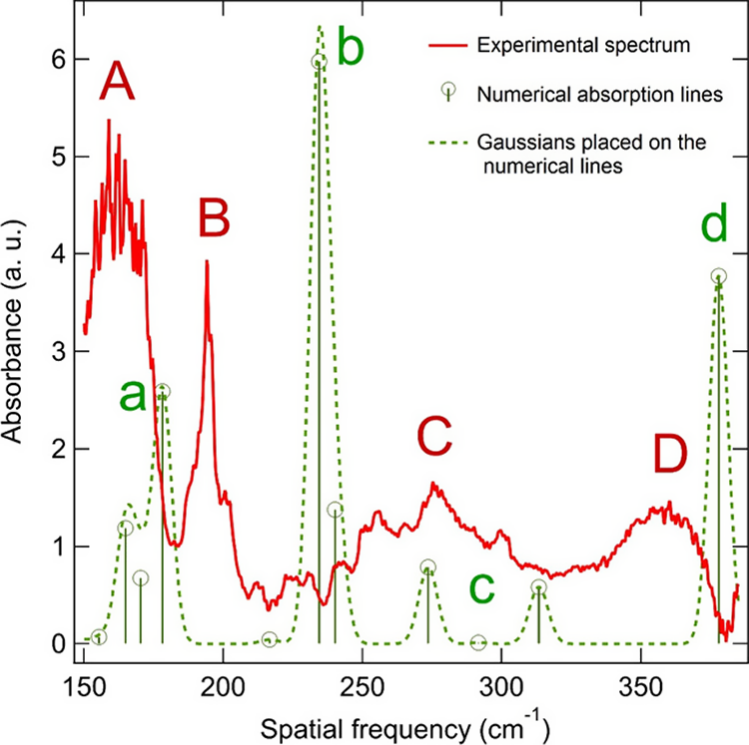
Linarite spectrum measured at 7.5 K for the electrical radiation field at 45° to the crystalline *a*-axis for light propagating along the *c*-axis (Figure 1d). The absorption bands are indicated by letters A–D. Round symbols (sticks to zero) show the intensity and energy of the spectrum obtained from the DFT modeling. Gaussian forms are drawn to these spectral lines as a guide to the eye to make the comparison with the experimental spectrum easier. The modeled bands are designated by letters a–d (in green), which correspond to the experimental bands A–D (in red).

**Figure 5:**
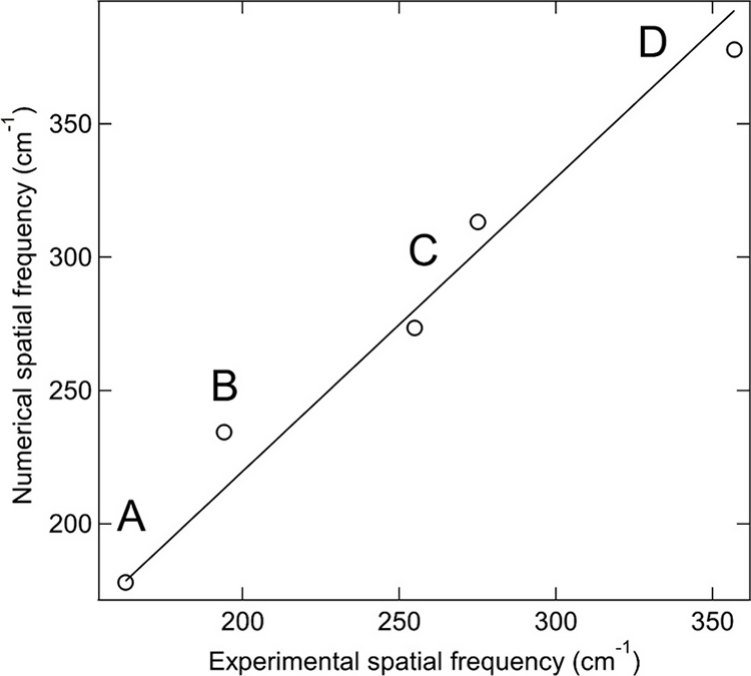
Correspondence between the energy (i.e., spatial frequency) of the experimental and modeled absorption bands of linarite. The solid line is the fit to the data, with a gradient of 1.1 ± 0.1.

Figure 4 shows this spectrum of linarite measured at 7.5 K, with an electric field at 45° to the *a*-axis (with THz wave propagation along the *c*-axis). This polarization is most suitable for comparison with the DFT modeled spectra, as it exhibits the combined modes for both polarization directions. However, the band intensity in the data is not expected to correspond to the calculated one for this very reason, that our DFT modeling does not account for the polarization of incoming light. Four main absorption bands can be identified in the experimental spectrum, centered at 162 (A), 194 (B), 275 (C), and 357 (D) cm^–1^. The corresponding modeled absorption lines are shown by round symbols. Gaussian profiles are centered at each of the modeled absorption lines, with Gaussian half-widths (all of 8 cm^–1^) which were chosen for the best correspondence with the experimental bands. The resulting modeled bands are labeled as a–d so that they correspond to the experimental bands labeled A–D. The modeled spectrum implies that the experimental bands contain contributions from more than one phonon mode. The broad band observed at C corresponds to several distinct modeled lines, which did not merge when assuming the Gaussian half-width of 8 cm^–1^ for all modeled modes. Figure 5 collates the correspondence between the central energy (i.e., spatial frequency) of the experimental bands (red lines in Figure 4) and the corresponding dominant modeled lines (green lines in Figure 4). There is a good correlation between the two. The solid line in Figure 5 is the fit to the data, giving a straight line with a gradient of 1.1 ± 0.1. Therefore, the energy of the modeled absorption lines is on average 10% higher than the energy of the corresponding experimental bands. This is seen in Figure 4 as a shift of the modeled spectrum to the right with respect to the experimental spectrum. This shift is expected to occur for the DFT modeling because of the approximations used,^34^ which include the DFT functional, the degree of the geometry convergence, the pseudopotential, the cutoff energies, and the calculation of phonon modes within a harmonic approximation. The modeled intensities of the absorption bands roughly agree with the experimental intensities, i.e., intense/weak experimental bands have corresponding intense/weak modeled lines. The disagreements occur not only because of the approximations used but also because the modeling does not account for the peak broadening, thermal effects, and the polarization of the incoming terahertz beam. Experimental intensity can also suffer from uneven sensitivity of the setup in the measured frequency range, distorting the intensities in various parts of the spectrum. Overall, the modeled spectrum is accurate enough to enable assignment of the modeled modes to the experimental absorption bands.

Table 1 describes the normal modes of vibration assigned to each of the experimental bands in Figure 4. The Supporting Information contains the visualization file for these and other normal modes. Most of the experimental bands consist of merged sub-bands belonging to each of the normal modes. All the bands can be divided into 3 groups, where prevalent vibrations occur within the plains containing either PbSO_4_ chains, Cu(OH)_2_, or both. Band A is associated with vibrations of both PbSO_4_ and Cu(OH)_2_ (Table 1). Band B is associated with the vibration of PbSO_4_, while bands C and D are associated with the vibration of Cu(OH)_2_. The only exception to this is the mode at 164.81 cm^–1^; however, this is not the dominant mode contributing to band A. Modes at 155.32, 216.63, and 291.65 cm^–1^ were calculated to have an insignificant contribution to the infrared spectrum.

**Table 1:** Description of Normal Modes of Vibration, As Obtained by DFT Modeling, between 150 and 400 cm^–1^

wavenumber (cm^–1^)	assigned experimental band	vibration description		IR activity from DFT modeling
		PbSO_4_ chains	Cu(OH)_2_ planes	
155.32		in-plane twisting of SO_4_; stretching of Pb–O bonds; symmetric between planes; Pb stationary	stationary	very weak
164.81	A	in-plane twisting of SO_4_; stretching of Pb–O bonds; antisymmetric between planes; Pb stationary	stationary	Y
170.27	A	in-plane twisting of SO_4_ and of Pb–O bonds in half of PbSO4 chains; Pb stationary	in-plane twisting of Cu(OH)_2_, symmetric between PbSO_4_ and Cu(OH)_2_ layers	Y
178.13	A	in-plane twisting of SO_4_ and of Pb–O bonds in half of PbSO_4_ chains; Pb stationary	in-plane twisting of Cu(OH)_2_, antisymmetric between PbSO_4_ and Cu(OH)_2_ layers	Y
216.63		out-of-plane twisting of SO_4_, symmetric within PbSO_4_ chain layers; stretching of Pb–O bonds; Pb stationary	stationary	very weak
234.43	B	out-of-plane twisting of SO_4_, antisymmetric within PbSO_4_ chain layers; stretching of Pb–O bonds; Pb stationary	stationary	Y
240.13	B	out-of-plane twisting of SO_4_, antisymmetric within PbSO_4_ chain layers, antisymmetric between PbSO_4_ and Cu(OH)_2_ layers; stretching of Pb–O bonds; Pb stationary	stationary	Y
273.58	C	almost stationary	in-plane stretching of Cu(OH)_2_ planes	Y
291.65		out-of-plane twisting of SO_4_; stretching of Pb–O bonds; Pb stationary	stationary	very weak
313.3	C	almost stationary	out-of-plane bending of Cu(OH)_2_ planes	Y
377.92	D	almost stationary	out-of-plane bending of Cu(OH)_2_ planes	Y

Each PbSO_4_ plane consists of two subplanes made up of chains that are mutually connected through weak van der Waals bonds, rather than hydrogen bonds (Figure 1a). They are also connected to the Cu(OH)_2_ planes through interplanar hydrogen bonds. The Cu(OH)_2_ chains are mutually connected through hydrogen bonds within their own plane. Because thermal expansion is strongest along the weak van der Waals bonds between the PbSO_4_ planes, the absorption bands associated with the PbSO_4_ planes are expected to have a stronger temperature dependence than the bands associated with the Cu(OH)_2_ planes. Experimental data (Figure 2) show that the higher energy bands, labeled as bands C and D in Figure 4, have the weakest intensity and band B has the strongest temperature dependence of intensity. This gives strong support to our assignment of the absorption bands in Figure 4. This is described in detail within the Supporting Information through Figures S1–S6.

The modeled bands as plotted in Figure 4 are taken as the average of all of the closely spaced calculated modes. From Figure 4, there are a couple of modeled bands that are infrared-silent, i.e., with zero absorption intensity expected at 0 K. These are located within the b- and c-numerical bands at around 216 and 291 cm^–1^. However, in contrast to this, we observe infrared absorption intensity in the experimental band C, and somewhat less in band B, indicating an enhancement of the modes that are expected to be infrared-silent. A likely reason for this is that the spectrum in Figure 4 is measured with *E* of the incoming synchrotron beam at 45° to the *a*-axis, while the occurrence of the infrared-silent mode at low temperature is observed only for *E* || *a* (Figure 2a). Furthermore, defects in the natural crystal samples or thermal enhancement of hot modes due to local symmetry breaking may also play a role, as discussed earlier.

**Figure 6:**
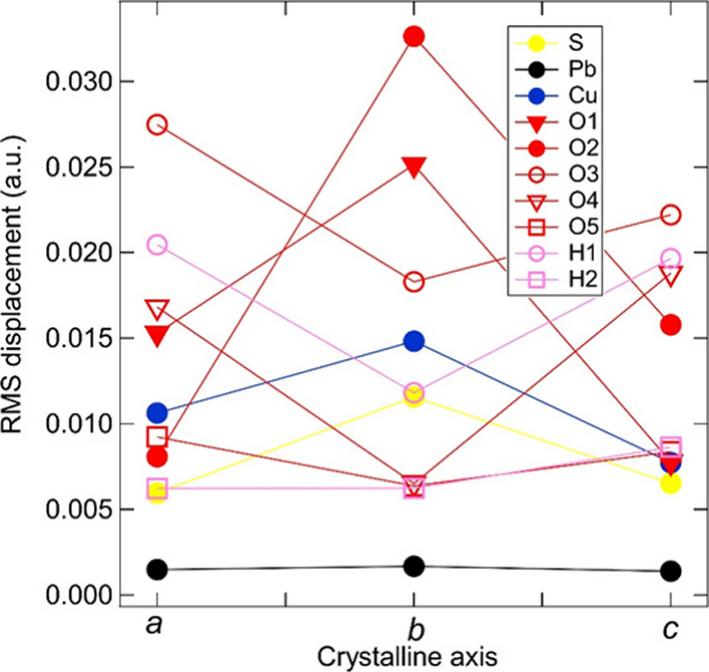
Modeled vibrational amplitudes projected onto each of the crystalline axes for each of the atoms in the unit cell, averaged over all phonon modes accessed in the experiment, with energies between 150 and 400 cm^–1^ (Figure 2). The numbering of oxygen and hydrogen atoms is consistent with the Wyckoff designation of atomic sites for this unit cell.

The vibrational phonon modes excited by the synchrotron radiation could be correlated to the growth morphology of linarite and thus to the crystal axes of linarite. In fact, the anisotropic vibrational amplitude of each atom in the unit cell may hold the key to subtle local distortions in the crystal structure responsible for occurrence of infrared modes as the temperature increases at energies where only Raman modes are expected, i.e., the occurrence of hot bands. To test this hypothesis, we calculated the vibrational amplitudes for each of the atoms in their respective Wyckoff positions in the unit cell along each of the crystalline axes and averaged over all phonon modes between 150 and 400 cm^–1^, as shown in Figure 6. Lead is by far the heaviest atom in the unit cell, and its vibrational amplitudes were the lowest, as expected. Copper, sulfur, and lead all have their largest average vibrational amplitudes along the crystalline *b*-axis (Figure 6), while the amplitudes along the *a*- and *c*-axes are similar to each other. Due to their occupation of different Wyckoff positions with different local symmetries in the unit cell, the vibrational amplitudes for the oxygen and hydrogen atoms are more varied. Here, the vibrational amplitudes along the *b*-axis are at maximum for the O1 and O2 sites but are at minimum for the O3, O4, and O5 sites, as well as for the two hydrogen sites. Interestingly, the largest displacement of all of the atomic positions occurs for the O2 sites along the *b*-axis. As depicted in Figure 1a, this corresponds to the vertex of the SO_4_ tetrahedra located in the void between the CuO_4_ chains and Pb ions stacked along the *a-*axis. This suggests significant tilting or twisting of the SO_4_ tetrahedra along the *b*-axis due to the lattice dynamics in the 150–400 cm^–1^ energy range. Because anharmonic effects are more likely to occur in vibrations with the largest atomic displacements, we propose that the vibrations of SO_4_ tetrahedra drive the symmetry breaking and trigger the observed hot band at 230 cm^–1^ in linarite.

Having described the vibrational modes in Table 1, we now attempt to identify the mode associated with absorption band #3 in Figure 2a. This band shows evidence of coupling with the ∼230 cm^–1^ hot band at high temperatures and thus may be related to the corresponding distortions that break local inversion symmetry, allowing infrared activity of a Raman mode. Following the above argument, it is therefore tempting to assign band #3 (observed at ∼290 cm^–1^) to the motion of the SO_4_ tetrahedra. Although these are excited by *E* || *a* polarization, they should also feature a significant displacement along the crystalline *b*-axis. Only two vibrational modes with such characteristics were obtained in the vicinity of 290 cm^–1^ in our modeling (Table 1): one at 240.13 cm^–1^ and the other at 291.65 cm^–1^. Considering that the energies of the vibrational modes were calculated in the harmonic approximation, their values are expected to be higher than the energies of the corresponding experimental absorption bands. Thus, we identify the mode at 291.65 cm^–1^ as a candidate for the observed band #3 in Figure 2a. Assigning the vibrational mode for the Raman band at 230 cm^–1^ that becomes infrared-active at elevated temperatures (i.e., a hot band) through thermal excitation of band #3 is more difficult, since the infrared intensities obtained in DFT modeling are not very accurate. The mode at 216.63 cm^–1^ is a potential candidate, and its infrared activity is very low. An alternative option might be the mode at 273.58 cm^–1^, which is infrared-active in our modeling. Indeed, this mode is at about the right energy, considering that the modeled energies are on average about 10% higher than the experimental ones (Figure 5). The remaining modes around these energies have substantial infrared absorption and can probably be discarded as potential candidates, being assigned to band B (Figure 4).

To recapitulate, our original aim was to determine the interplay between the structure and magnetism in linarite and to look for a link between structural excitations and low-dimensional magnetism. From the THz spectroscopic measurements, we did not observe any magnetic excitations on the energy and temperature scales accessible to us. Given that the magnetic-excitation energy scales are extremely low^4,5^ and originate from very small Cu magnetic moments, it is possible that we did not have access to these regions in our experiments. However, our observations using polarized THz spectroscopy, together with DFT modeling, have allowed us to conclude that the vibrational motion for phonon modes in the 150–400 cm^–1^ range is highly anisotropic. The vibrational amplitudes along the crystallographic *b*-axis take on their maximum amplitude (or minimum for some hydrogen and oxygen atoms) for all atoms in the unit cell. These results are coincidental with the anisotropy implied by the 2-fold screw axis along the *b* crystallographic direction, which is also the direction of the needle-like crystal growth, the strongest vibrational amplitude orientation, the strongest magnetic interaction direction, and the most likely axis for which a structural distortion to *P2*_*1*_ symmetry can take place. This suggests the potential for spin–phonon coupling in linarite as a way of stabilizing the coupling within the quasi-1D Cu–O chains, which in turn allows for the observation of novel magnetic states both in zero and applied fields.^4^ There is some correlation between these three metrics, morphological growth, vibrational amplitude of phonon modes, and magnetic interaction strength, which may help to promote the influence of spin–phonon coupling in this material. Similarly, the presence of an anomalous hot band in the region of 230 cm^–1^ for *E* || *a* radiation might be attributed to a Raman-active mode becoming IR-active as a result of anharmonic effects and local symmetry breaking. Although linarite does not undergo a global structural distortion below 300 K, local structural deviations concerning the O1 and O2 sites that contribute to a tilting of the SO_4_ tetrahedra along the *b*-axis at high temperatures can break the local inversion symmetry and may be likened to other subtle distortions observed in similar materials.^10,32,33^ To better understand the hot-mode origin as well as any corresponding local or incommensurate structural distortions, a detailed inelastic X-ray or neutron study would be useful to probe the full phonon dispersion, in particular, to identify any incommensurate or short-range soft mode transitions that may occur at high temperatures. We hope that the current work will motivate such further studies. Revealing the details of the magneto-structural correlations in linarite may also pinpoint the mechanism of the ferroelectric behavior normal to the *b*-axis below the magnetic transition in linarite; however, this is a topic for future investigations.

## Conclusions

A polarized THz spectroscopic study has been carried out on a single crystal of naturally grown linarite, PbCuSO_4_(OH)_2_. The experiment has been accompanied by DFT modeling. Experimental results gave a strongly polarization-dependent phonon spectrum in the energy range of 150–400 cm^–1^. The observed phonon absorption bands were assigned through DFT modeling. The assignment highlighted how the vibrational amplitudes along the crystallographic *b*-axis take on either maximum or minimum values relative to the *a*- and *c*-axes for all atoms in the unit cell. This strong vibrational anisotropy along the *b*-axis hints at a significant connection between the structure of linarite and its magnetism: vibrational amplitudes are determined by the bonds in the crystal, while the magnetism of linarite arises from the CuO chains which propagate along the *b*-axis and contain the strongest magnetic exchange interactions. Furthermore, an anomalous “hot-mode” absorption band may be a signature of local symmetry breaking at higher temperatures involving subtle distortions due to tilting of the SO_4_ tetrahedra along the *b-*axis. This points to the potential for significant magneto-structural correlations in linarite. We hope that our work will motivate a more thorough investigation of this coupling.

## Supporting Information Available

Additional information regarding the observed resonant modes in linarite and fits to the THz data for the electric field parallel to both the *a* and *b* crystallographic axes; additional information comparing the observed hot band with a normal band and the low energy, low temperature, and applied field data collected for linarite in the magnetically ordered phase; and a link to the DFT modeling .mold file (PDF)
