# Supplementary material for: Terahertz Vibrational Dynamics and DFT Calculations for the Quantum Spin Chain Linarite, PbCuSO4(OH)2
Source: J Phys Chem A. 2024 Feb 28;128(10):1767–75. doi: 10.1021/acs.jpca.3c06926 (PMC10945475; doi:10.1021/acs.jpca.3c06926)
Supplement: Supplementary file 1 — jp3c06926_si_011.pdf [file jp3c06926_si_011.pdf]

Supporting Information for: Terahertz Vibrational Dynamics and DFT Calculations for the Quantum Spin Chain Linarite, $PbCuSO_4(OH)_2$

*Andrew Squires[1,2], Evan Constable[1,3], Joseph Horvat[1], Dominique Appadoo[4], Ruth Plathe[4], R. A. Lewis[1], Kirrily C. Rule[1,5]**

1) University of Wollongong, Wollongong NSW 2522, Australia

2) Commonwealth Scientific and Industrial Research Organisation (CSIRO), Lindfield, NSW, 2070, Australia

3) Institute of Solid State Physics, TU Wien, 1040 Vienna, Austria

4) Australian Synchrotron, ANSTO, 800 Blackburn Rd Clayton, VIC 3168, Australia

5) Australian Centre for Neutron Scattering, ANSTO, Lucas Heights NSW 2234, Australia

SUPPLEMENTARY SECTION 1: SUMMARY OF OBSERVED RESONANT MODES IN LINARITE AT THZ FREQUENCIES

Supplementary Table S1 displays a summary of the energies and corresponding temperature dependence for the resonant modes presented in Fig. 2 in the main text. The resonant position/energy is presented at 15K, corresponding to the measurement closest to the theoretical modelling temperature of 0K. Further, this measurement provides the least thermal noise and highest accuracy in the fitting model employed.

Supplementary Table S1: Summary of resonant energy temperature dependence.

Incident **E** Polarisation	Peak number	Wavenumber at 15K (cm^{-1})	Energy at 15K (meV)	dE/dT
Main text Fig. 2(a)				
**E**‖a	1	186	23	0.024
**E**‖a	2	195	24	0.016
**E**‖a	3	290	36	-0.0015

Main text Fig. 2(b)				
E‖b	4	230	29	-0.16
E‖b	5	260	32	0.0019
E‖b	6	275	34	0.0099
E‖b	7	286	35	0.011

Fitting of the THz spectra was performed using the Multi-peak Fitting 2.0 tool in the proprietary software, Igor Pro 8. Absorption peaks were fitted with Gaussian curves superimposed on a linear baseline minimizing the effect of non-resonant material absorption. The fitting process was repeated sequentially for measurements from 15K upwards, until the software could no longer reliably identify the resonant features from thermal noise/population of modes. For **E** ‖ **a** and **E** ‖ **b** this condition occurs at 200K and 130K respectively,

SUPPLEMENTARY SECTION 2: FITTED DATA FOR INCIDENT ELECTRIC FIELD PARALLEL TO THE A CRYSTALLOGRAPHIC AXIS

Supplementary Figures S1-S3 present the temperature dependence of the peak position (energy), amplitude and area respectively for **E ǁ a**. In Fig.S1, a relatively strong temperature dependence is observed for mode 1 and 2 (186cm^{-1} and 195cm^{-1} at 15K). That is, a blue shift with increasing temperature. These modes correspond to the B band(s) from the DFT model, which suggests these motions are attributed to the $PbSO_4$ planes. As such, they are expected to exhibit a large energy dependence with temperature, as observed. This provides good evidence in attributing these modes to this band in the model. A summary of the energy temperature dependence of all modes can be found Supplementary Table S1.

Further, these planes are expected to have the highest intensity irrespective of the THz polarization. This is also reflected in Fig. S2, with the amplitude(s) consistently more than double that of any other peak in both polarizations, except the 290cm^{-1} in **E ǁ a** (peak 3)

It should be noted that in Fig. S2, the rise in amplitude for peak 1 from 50-100cm^{-1} should be interpreted with caution. While the numerical fitting could reliably distinguish these features as standalone in position up to 140K, the profiles of each peak overlapped significantly leading to struggles in accurately producing their true absorption. Above 80K, the signal became too weak to easily extract the peaks in the fit. Further, frequency shifting in each modes moved them closer

together. Above 140K only 1 peak could now be reliably extracted in the fit (hence the cut-off in the peak 2 data). As such, the amplitude data for peak 1 likely contains an input from peak 2 above 50K and contains all the residual peak 2 contribution above 140K.

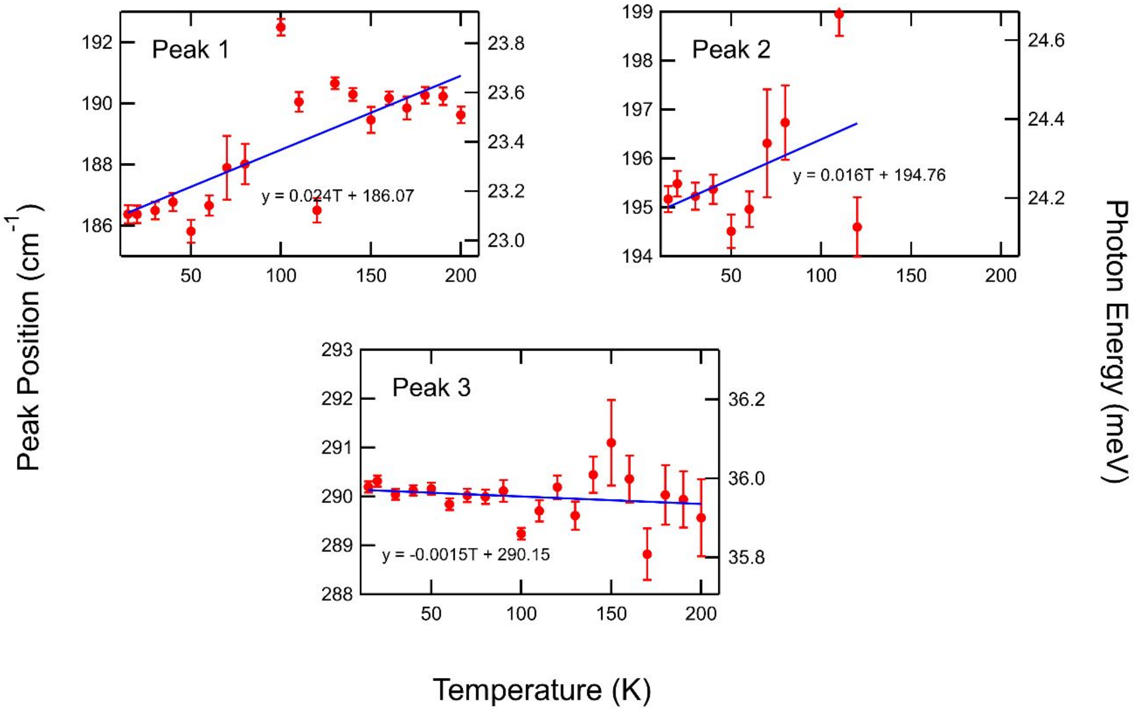

Supplementary Figure S1. Resonant energy temperature dependence for **E** ‖ **a**: Blue lines show a linear fit of the plotted (red) data.

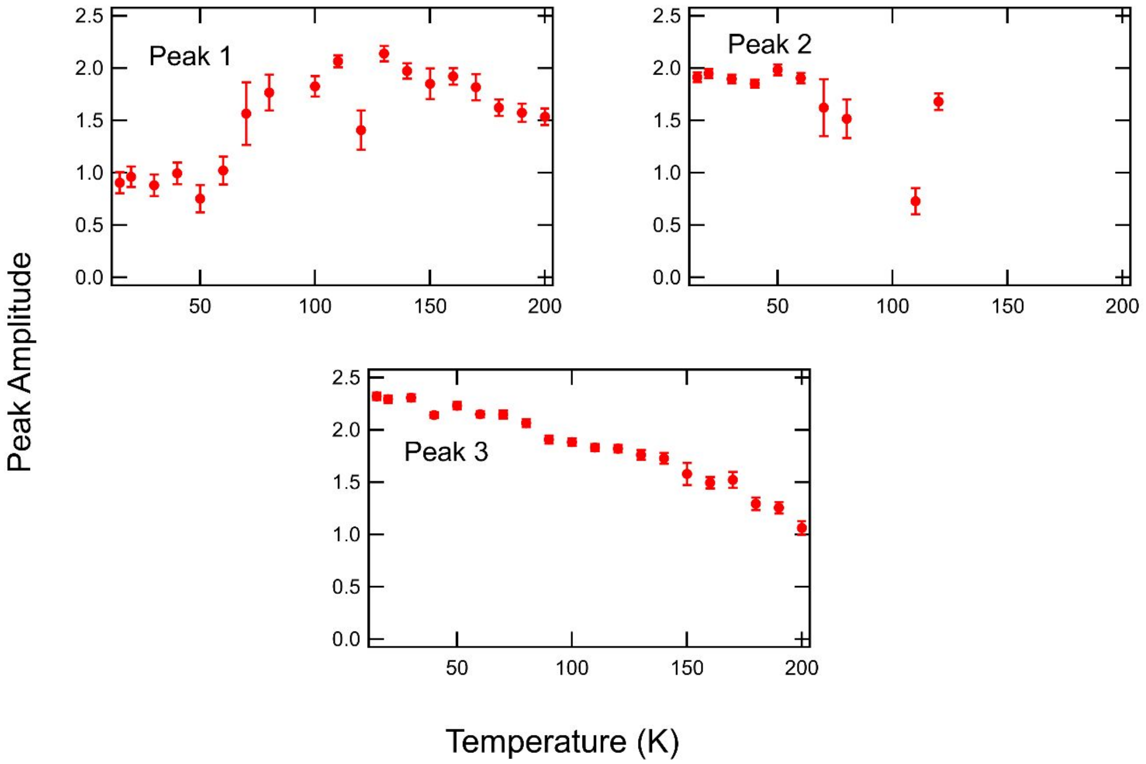

Supplementary Figure S2. Peak amplitude temperature dependence for **E ∥ a**

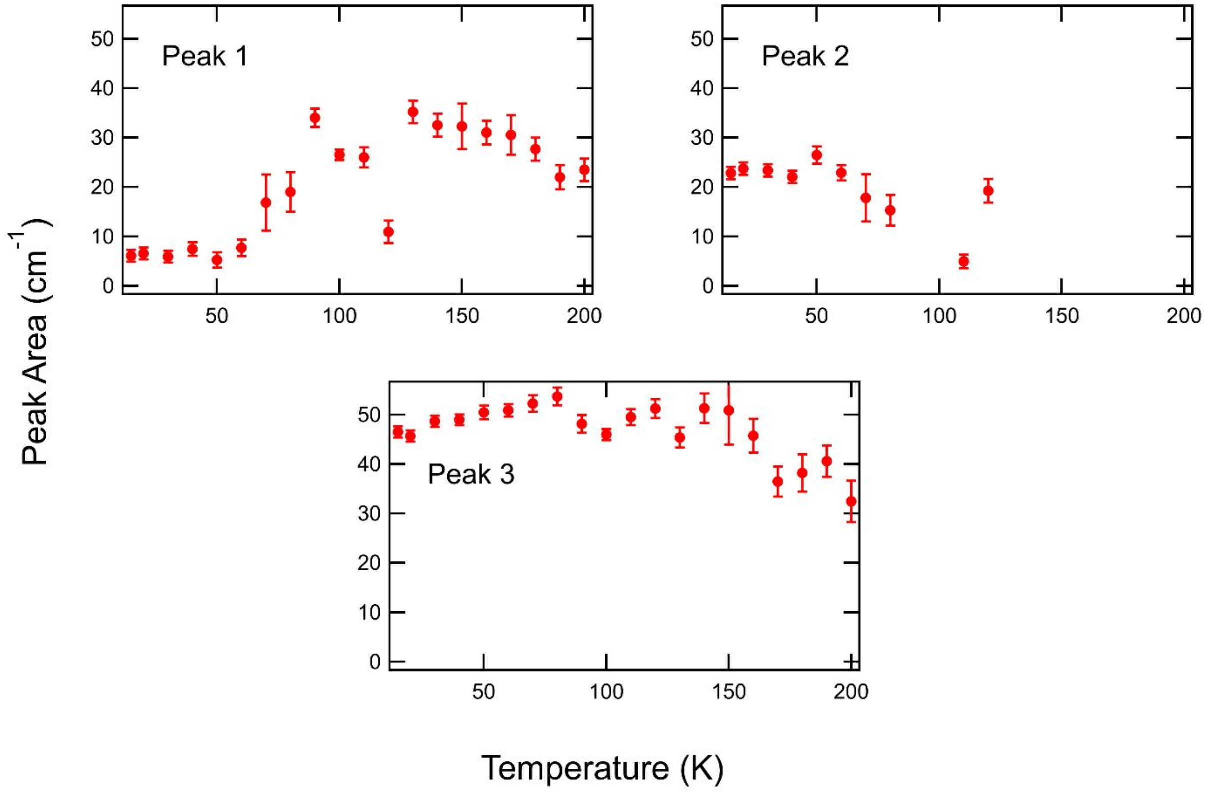

Supplementary Figure S3. Peak area temperature dependence for **E ∥ a**

SUPPLEMENTARY SECTION 3: FITTED DATA FOR INCIDENT ELECTRIC FIELD PARALLEL TO THE B CRYSTALLOGRAPHIC AXIS

Supplementary Figures S4-S6 present the temperature dependence of the peak position (energy), amplitude and area respectively for **E** || **b**. Interestingly in Figure S4, peak 4 exhibits a relatively strong red shift with temperature. Peaks 5-7 show weaker blue-shifts compared to those in Figure S1. These weaker shifts are consistent with the $Cu(OH)_2$ planes as predicted from the C and D bands in the DFT model. Further, they show a much lower resonant amplitude (Fig. S5) (0.2 – 1) than those in Fig. S2 (1-2) for the $PbSO_4$ planes. This gives further evidence in the assignment of these modes.

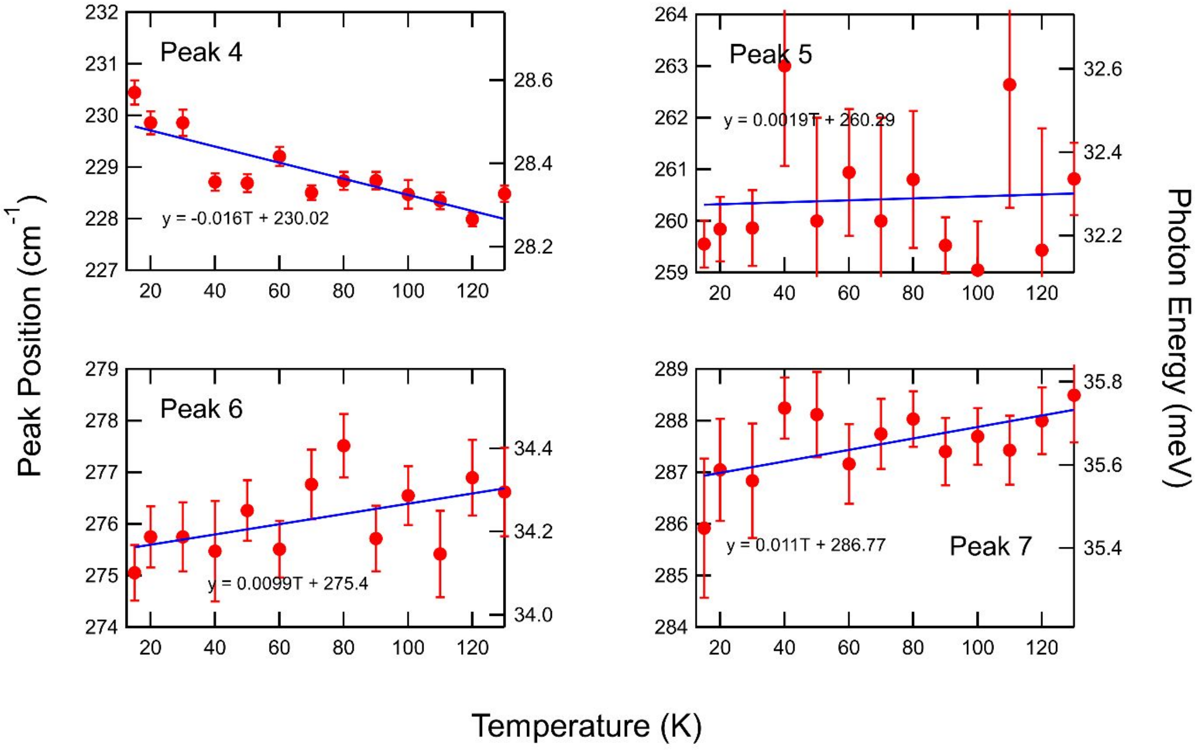

Supplementary Figure S4. Resonant energy temperature dependence for **E** ∥ **b**: Blue lines show a linear fit of the plotted (red) data

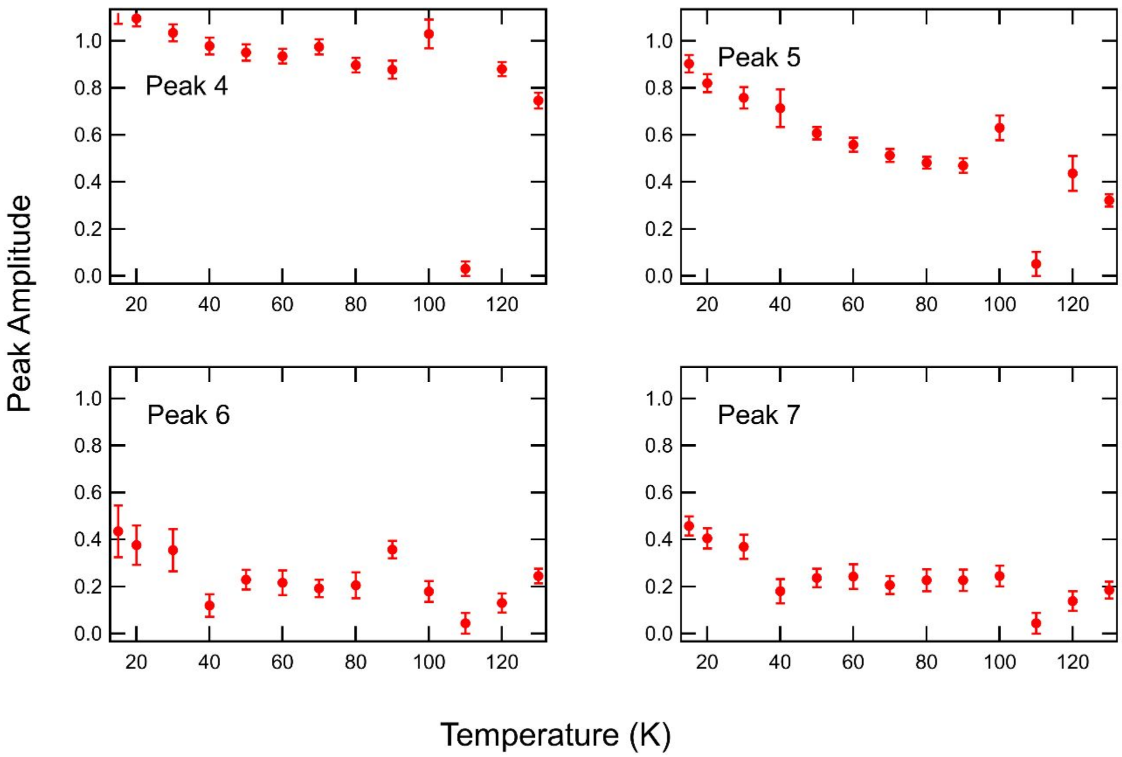

Supplementary Figure S5. Peak amplitude temperature dependence for **E ∥ b**

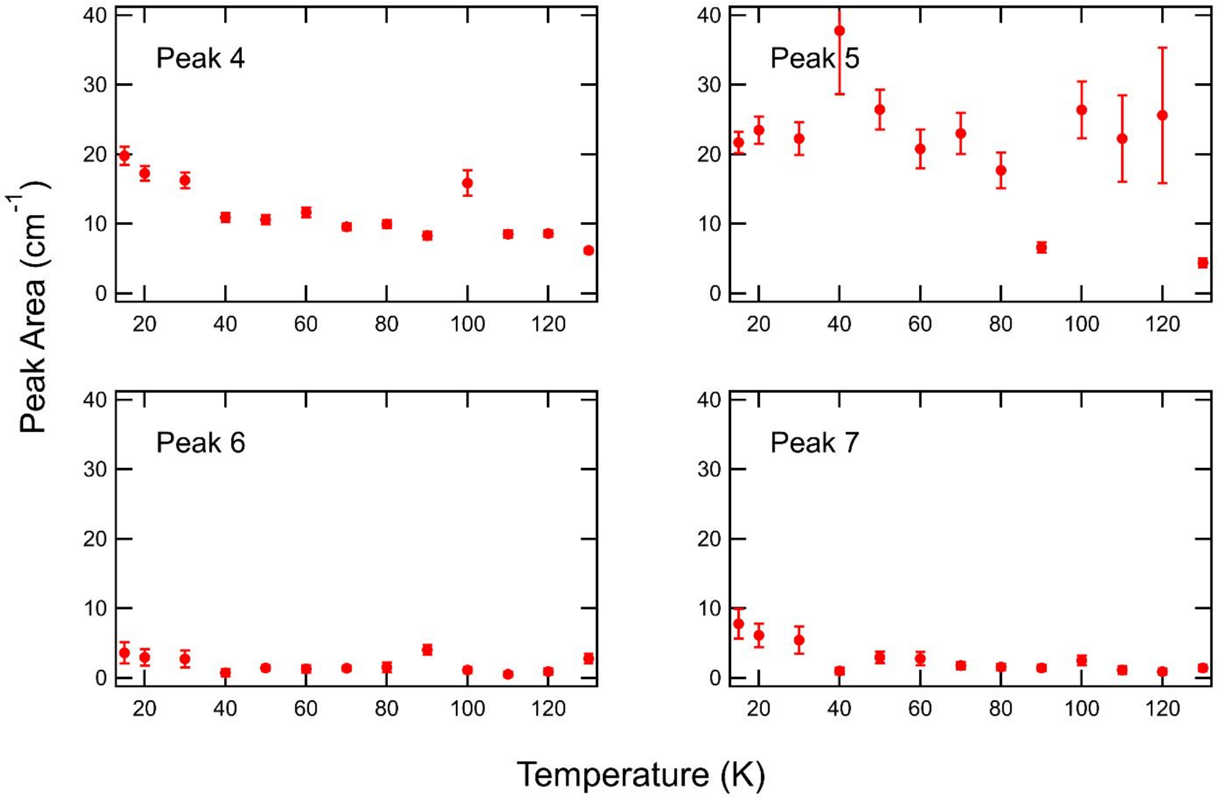

Supplementary Figure S6. Peak area temperature dependence for **E || b**

SUPPLEMENTARY SECTION 4: FITTED DATA USED TO CALCULATE THE INTERPLAY OF THE SPECTRAL WEIGHT BETWEEN PEAK 3 AND THE HOT BAND

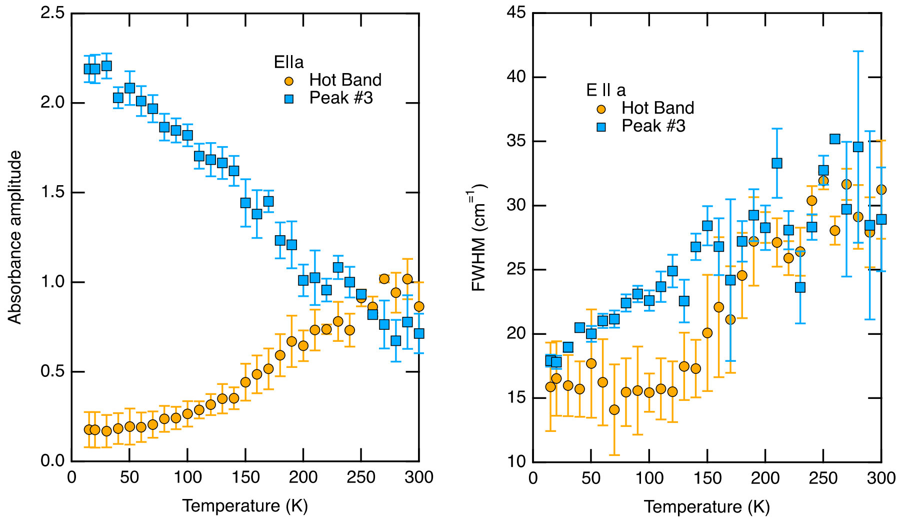

Supplementary Figure S7. Comparison of the amplitude and FWHM of peak 3 with the **E || a** hot band

Fig. S7 depicts the amplitude dependence and FWHM of the **E || a** hot band and peak 3 with temperature. The spectral weight in the main text Fig. 3 was calculated as the product of this data

with the resonant frequency. It should be noted there is a subtle difference in the peak 3 amplitude here with that presented in Fig. S2. Higher attention was placed on the fitting for this mode, accounting for possible variation in the spectral baseline. Multiple fits were performed on this peak standalone, with differing baselines and peak profiles. The Fig. S7 amplitude and FWHM is a statistical average of these fits, whereas the data in Fig. S2 and S3 is from a single fit encompassing all the peaks 1-3 with a single linear baseline.

SUPPLEMENTARY SECTION 5: LOW FREQUENCY (20 – 90cm^{-1}) SPECTRAL INFORMATION FOR LINARITE

Fig. S8 shows spectra taken on a FTIR spectrometer with a Hg lamp source at the University of Wollongong for **E** || **a** and **E** || **b** optical configurations. The results reveal several weak absorption bands in this energy range. None of the features are affected by applied magnetic field along either the *b* or *a* (not shown) directions. While the absorption in the **E** || **a** configuration appears to be phononic in nature (although this is low energy for phonons it is not beyond the realm of possibility) the feature in the **E** || **b** is rather unusual. It shows a continuum like profile with a sharp edge at ~65 cm^{-1} followed by another broad absorption band above that, likely attributable to the

lowest energy absorption band shown for this polarization in the main text. This continuum feature only seems to appear at low temperatures, but unfortunately, we did not track its temperature dependence carefully at the time of measurement. It does not seem to be affected by applied magnetic fields up to 5 T along both the *a* and *b* crystal directions. Moreover, it is still present at 5 K where the non-collinear magnetic order is supposed to be suppressed.

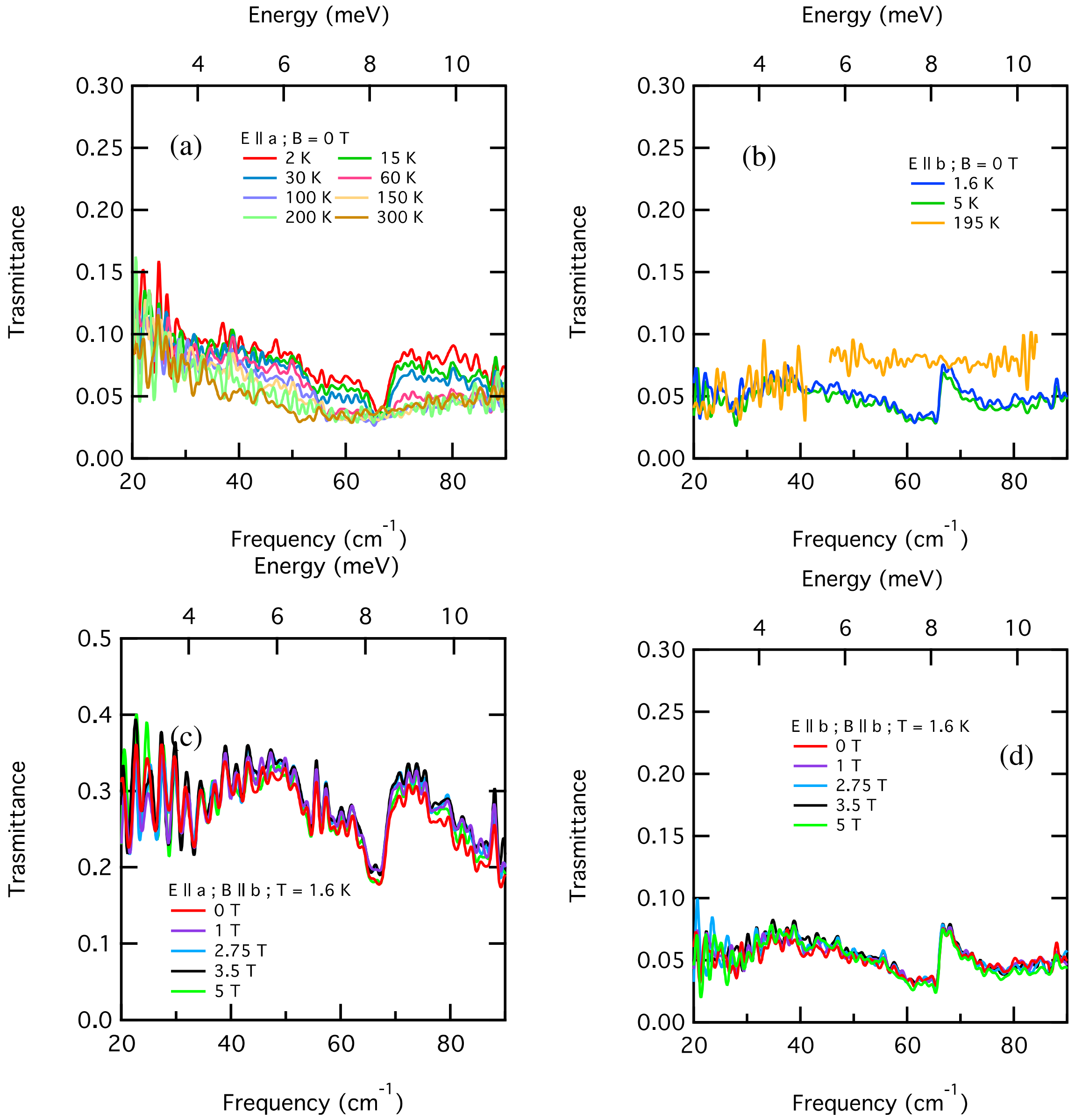

Supplementary Figure S8. Temperature dependent transmission spectra between 20-90 cm^{-1} for **E** || **a** (a) and **E** || **b** (b). Transmission dependence on applied magnetic field along the *b* direction for **E** || **a** (c) and **E** || **b** (d) at ~1.6 K.

SUPPLEMENTARY SECTION 6: DFT SIMULATIONS OF VIBRATIONAL DYNAMICS

As part of the DFT modelling a .mold file was produced (linarite_modes_ALL.MOLD), which shows visualizations of each of the generated modes. Fig. S9 shows all vibrational modes in the calculated absorption spectrum of linarite.

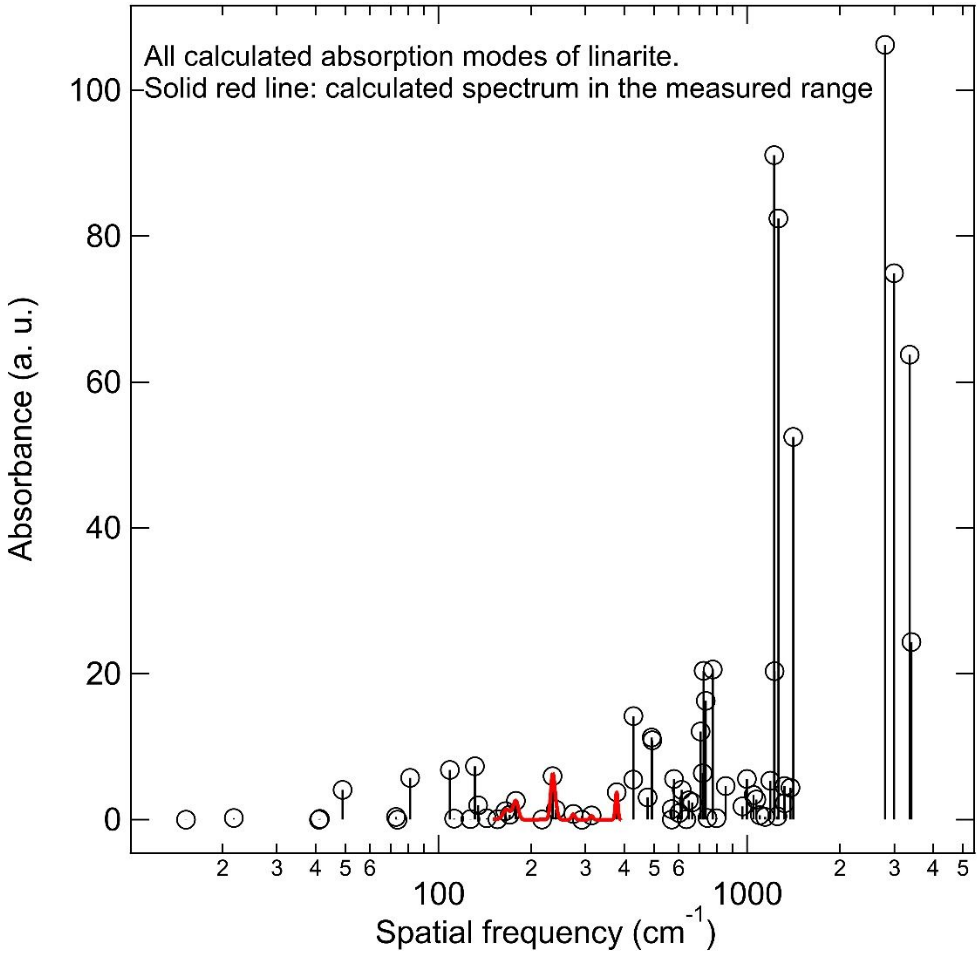

Supplementary Figure S9: Calculated absorption spectrum of linarite with all obtained absorption modes, with no spectral broadening. Solid red line shows the calculated spectrum in the spatial frequency range of our experiment. This line is the same as the calculated spectrum in Fig. 4 of the main text, where the spectral broadening is arbitrarily assigned so that the best agreement with the experiment is obtained.
